# Obesity as a Risk Factor for Hyperglycemia, Electrolyte Disturbances, and Acute Kidney Injury in the Emergency Department

**DOI:** 10.3390/biomedicines13020349

**Published:** 2025-02-03

**Authors:** Iulia Najette Crintea, Alexandru Cristian Cindrea, Ovidiu Alexandru Mederle, Teodor Florin Fulga, Adina Maria Marza, Alina Petrica, Cosmin Iosif Trebuian, Romulus Timar

**Affiliations:** 1 Department of Surgery, “Victor Babes” University of Medicine and Pharmacy, 300041 Timisoara, Romania; iulia.crintea@umft.ro (I.N.C.); alexandru.cindrea@umft.ro (A.C.C.); marza.adina@umft.ro (A.M.M.); alina.petrica@umft.ro (A.P.); trebuian.cosmin@umft.ro (C.I.T.); 2 Emergency Department, Emergency Clinical Municipal Hospital, 300079 Timisoara, Romania; 3 Faculty of Cybernetics, Statistics and Economic Informatics, The Bucharest University of Economic Studies, 010374 Bucharest, Romania; teoflorin02@gmail.com; 4 Emergency Department, “Pius Brinzeu” Emergency Clinical County Hospital, 300736 Timisoara, Romania; 5 Department of Anesthesia and Intensive Care, Emergency County Hospital, 320210 Resita, Romania; 6 “Pius Brinzeu” Emergency County Hospital, 300723 Timisoara, Romania; timar.romulus@umft.ro; 7 Second Department of Internal Medicine, “Victor Babes” University of Medicine and Pharmacy, 300041 Timisoara, Romania

**Keywords:** cardiovascular markers, metabolic markers, metabolic crises, obesity

## Abstract

**Background/Objectives**: Obesity is a global health challenge linked to a higher risk of metabolic and cardiovascular complications. This study investigates the role of cardiovascular markers in predicting metabolic crises in obese patients, focusing on the prevalence and clinical implications of these markers. **Methods**: This retrospective cohort study included 433 patients presenting with metabolic crises at the Emergency Department of Timișoara Municipal Emergency Hospital between 2019 and 2024. Patients were classified into obese (*n* = 161) and non-obese (*n* = 272) groups, with obesity further stratified into four grades based on body mass index (BMI). Cardiovascular markers, including NT-proBNP, troponin I, CRP, CK-MB, and D-dimer, alongside metabolic parameters, were analyzed. **Results**: Metabolic crises were significantly more prevalent in obese patients in all metabolic emergencies: hyperglycemia (27.9% vs. 11.0%, *p* < 0.001), electrolyte imbalance (23.6% vs. 9.2%, *p* < 0.001), and acute kidney injury (AKI) (12.4% vs. 5.5%, *p* = 0.01). NT-proBNP levels independently predicted AKI in obese patients (adjusted OR: 1.14 per 1000 pg/mL, 95% CI: 1.10–1.19, *p* < 0.001), with excellent discriminatory power (AUC: 0.88). Troponin I and D-dimer were higher in hyperglycemia and electrolyte imbalance, respectively, emphasizing the role of cardiac stress and pro-thrombotic states. Inflammatory markers such as CRP were significantly associated with metabolic disturbances, supporting the contribution of systemic inflammation. Comorbidities, particularly heart failure and atrial fibrillation, further increased the risk of metabolic crises. **Conclusions**: Cardiovascular markers suggest potential utility for early risk stratification of metabolic crises in obese patients. However, further studies are needed to validate their clinical applicability and to establish standardized approaches for integrating these biomarkers into routine practice, especially in patients with advanced obesity grades.

## 1. Introduction

Obesity has emerged as a critical public health challenge, with its prevalence doubling over the past four decades. Recent estimates indicate that almost 880 million adults and 159 million children worldwide suffer from obesity, reflecting a significant increase over previous decades [1]. This growing trend is associated with an increased risk of various metabolic disorders, particularly type 2 diabetes mellitus and cardiovascular disease. Among the severe manifestations of these metabolic disorders are acute metabolic crises, which require prompt medical intervention because they are life-threatening [2].

Obesity-related metabolic crises are driven by complex mechanisms, including chronic inflammation, insulin resistance, and endothelial dysfunction. Cardiovascular biomarkers provide measurable insights into these metabolic disturbances in obese individuals [3]. High-sensitivity C-reactive protein (hs-CRP) is an acute-phase protein whose increased levels indicate systemic inflammation and have been associated with increased cardiovascular risk [4]. Troponins, usually markers of myocardial injury, may be high in obesity due to subclinical heart strain or inflammation [5]. B-type natriuretic peptide (BNP) is released in response to ventricular volume expansion and pressure overload; higher BNP levels in obese patients may signal subclinical cardiac dysfunction [6].

The assessment of biomarkers, such as hs-CRP, can help predict acute metabolic crises by identifying individuals at higher risk. Elevated hs-CRP levels indicate an inflammatory state that may trigger metabolic decompensation, potentially leading to severe complications like diabetic ketoacidosis or hyperosmolar hyperglycemic state [7,8]. Similarly, higher levels of troponin and BNP could indicate underlying cardiac stress exacerbating metabolic instability. [9]. Incorporating the measurement of these biomarkers into clinical practice may improve early detection and facilitate timely therapeutic interventions for obese patients susceptible to metabolic crises [10].

Recent research has highlighted the complex interplay between obesity, systemic inflammation, and cardiovascular risk. Obesity is associated with increased levels of inflammatory markers, which are linked to components of the metabolic syndrome [11]. In addition, novel biomarkers, such as adipokines and cytokines, have been implicated in obesity-related metabolic complications, suggesting potential pathways for early detection and intervention [12]. Current clinical practices for monitoring obesity focus on traditional risk factors like blood pressure, lipids, and glucose but often fail to address the complex physiological changes in obesity, risking an underestimation of acute metabolic crisis potential [13]. Existing predictive tools for obese patients often lack accuracy, leading to delayed interventions. Integrating cardiovascular biomarkers such as hs-CRP, troponins, and BNP into routine assessments could improve risk stratification by revealing inflammatory and cardiac stress states [14]. By integrating these biomarkers, clinicians can identify high-risk patients earlier, enabling timely therapeutic interventions and potentially improving outcomes. However, there are currently no standardized guidelines for the use of these biomarkers in obese populations, highlighting the need for further research to establish their clinical utility and to develop comprehensive monitoring strategies that address the complicated nature of obesity-related health risks [15].

This study investigates the predictive role of cardiovascular biomarkers, including NT-proBNP, CRP, and D-dimer, in stratifying risks for metabolic crises among obese patients. By categorizing patients based on obesity grades, the research offers novel insights into the progressive impact of obesity severity on biomarker levels and their relationship with acute metabolic events. A key finding is the identification of NT-proBNP as an independent and robust predictor of acute kidney injury in obese individuals, providing new evidence for its clinical utility in early risk stratification and personalized management. These contributions aim to enhance understanding and clinical approaches to managing high-risk obese populations.

## 2. Materials and Methods

### 2.1. Study Design

This retrospective observational study was conducted at the Emergency Department (ED) of the Timișoara Municipal Emergency Hospital, Romania. The research includes patient data collected over a five-year period, from 1 January 2019 to 1 March 2024. The study was approved by the Institutional Review Board of the Timișoara Municipal Emergency Hospital (approval number: E-5945/5 November 2024). The design was chosen to utilize existing clinical data for identifying cardiovascular biomarkers predictive of metabolic crises in obese patients.

### 2.2. Study Population

**Figure 1:**
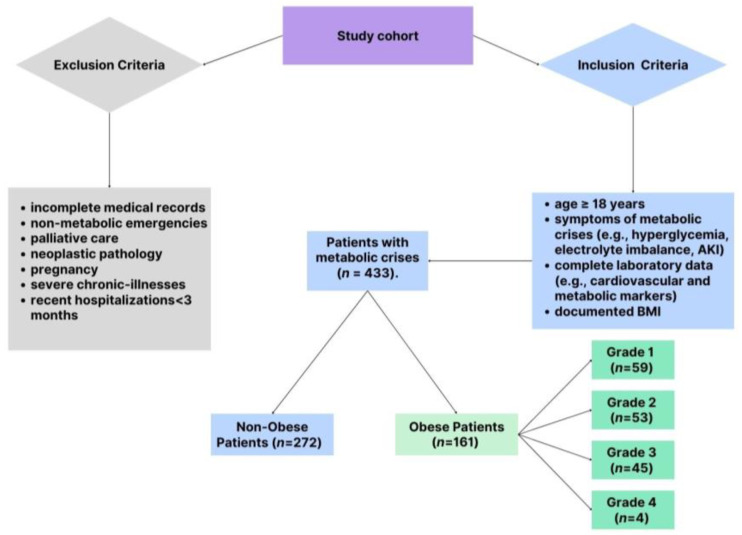
Patient selection.

The study included patients presenting to the ED with clinical features indicative of metabolic crises. To ensure a robust and representative cohort, specific criteria were applied for inclusion and exclusion, balancing the need for scientific rigor with clinical relevance. The patient selection process is shown in Figure 1, which details the inclusion and exclusion criteria applied to identify the final study cohort of 433 patients, which included 161 obese and 272 non-obese patients. Patients were eligible for the study if they were adults ≥18 years old presenting with documented clinical symptoms consistent with metabolic crises. These symptoms included significant electrolyte disturbances, such as hyperkalemia, hypokalemia, hyponatremia, or hypernatremia, as well as hyperglycemia characterized by blood glucose levels exceeding 250 mg/dL. Acute kidney injury was also a key criterion, defined as a serum creatinine increase of at least 0.3 mg/dL within 48 h or a 1.5-fold rise from baseline levels. Acute kidney injury was defined per the Kidney Disease Improving Global Outcomes (KDIGO) guidelines [16]. Patients with complete laboratory data, including cardiovascular biomarkers, were eligible. A total of 433 patients were included in the study, comprising 161 obese patients (BMI ≥30 kg/m^2^) and 272 non-obese patients (BMI <30 kg/m^2^). Obesity was determined using body mass index (BMI) thresholds established by the World Health Organization (WHO) [1]. Patients with a BMI of 30 kg/m^2^ or higher were categorized as obese. The severity of obesity was further stratified into four distinct grades to facilitate detailed analysis: Grade 1 (BMI ranging from 30.0 to 34.9 kg/m^2^), Grade 2 (BMI between 35.0 and 39.9 kg/m^2^), Grade 3 (BMI from 40.0 to 44.9 kg/m^2^), and Grade 4 (BMI of 45.0 kg/m^2^ or higher).

To maintain the focus of the study, several exclusion criteria were applied. Patients with incomplete medical records, particularly those missing critical demographic or laboratory data, were excluded. Also omitted were individuals presenting with non-metabolic emergencies such as trauma or surgical conditions, as well as patients receiving palliative care or those with neoplastic pathology. Given the unique metabolic and hormonal dynamics during pregnancy, pregnant women were not included in the study. Furthermore, patients with known advanced chronic illnesses, such as end-stage renal disease, severe heart failure, or active malignancies, were excluded to avoid confounding the analysis. Recent hospitalizations within three months for similar conditions were another exclusion criterion, as these could interfere with the interpretation of biomarker levels and outcomes.

### 2.3. Data Collection

Patient data were obtained from the electronic medical records of the Emergency Department at Timișoara Municipal Emergency Hospital. The dataset focused on cardiovascular biomarkers, including NT-proBNP, troponin I, and CK-MB, which are key indicators of cardiac stress and injury. Additional laboratory parameters, such as CRP, D-dimer levels, and basic hematological and metabolic markers, provided insights into inflammation and systemic complications. These biomarkers were analyzed in relation to the presence and severity of metabolic crises, stratified by obesity categories and comorbid conditions.

### 2.4. Parameters Assessed

Cardiovascular markers analyzed in this study included NT-proBNP, troponin I, CRP, CK-MB, and D-dimer. These were measured using standard laboratory techniques as part of routine clinical assessments in the Emergency Department. NT-proBNP (N-terminal pro-B-type natriuretic peptide): measured using a quantitative immunoassay to assess cardiac stress and ventricular wall tension. Troponin I: quantified using high-sensitivity assays to detect myocardial injury [17,18]. CRP (C-reactive protein): assessed as an indicator of systemic inflammation using a high-sensitivity method [19,20]. CK-MB (Creatine Kinase-MB): evaluated to identify myocardial stress [21]. D-dimer: measured to assess coagulation activity and the presence of a pro-thrombotic state [19].

In addition to these biomarkers, metabolic parameters such as glucose, potassium, sodium, urea, and creatinine levels were measured to evaluate metabolic crises. All tests were conducted using certified clinical laboratory methods in compliance with institutional protocols.

### 2.5. Impact of COVID-19 Pandemic on Patient Selection

The study was conducted during the COVID-19 pandemic; however, all patients included were confirmed COVID-19-negative prior to admission to the Emergency Department. Rapid antigen tests were systematically performed on all patients upon arrival, and those with positive results were directed to a separate dedicated COVID-19 treatment area and excluded from this study. This ensured that the population analyzed was not confounded by COVID-19-related metabolic or cardiovascular complications.

### 2.6. Statistical Analysis

Statistical analysis was conducted using MedCalc^®^ Statistical Software version 23.1.3 (MedCalc Software Ltd., Ostend, Belgium; https://www.medcalc.org; 2025). Continuous variables were presented as mean ± standard deviation (SD), while categorical variables were expressed as frequencies and percentages. Group comparisons were conducted as follows: continuous variables (comparisons between obese and non-obese groups were assessed using independent *t*-tests for normally distributed data, and the Mann–Whitney U test was applied for skewed data. Normality was tested using the Shapiro–Wilk test); categorical variables (these were compared using the Chi-square test or Fisher’s exact test for small sample sizes); analysis of variance (ANOVA to compare differences across obesity grades (Grade 1 to Grade 4), one-way ANOVA was used. Post-hoc pairwise comparisons were conducted using the Tukey HSD test to identify significant differences between specific obesity grades); multivariate logistic Regression (to evaluate the predictive role of cardiovascular biomarkers, multivariate logistic regression models were employed. These models adjusted for confounders such as age, sex, and comorbidities. Results were reported as odds ratios (OR) with 95% confidence intervals (CI). Model evaluation was performed by assessing the fit of logistic regression models using the Hosmer–Lemeshow goodness-of-fit test, while the discriminatory ability of the models was evaluated using the area under the receiver operating characteristic (ROC) curve (AUC)). A *p*-value of <0.05 was considered statistically significant for all analyses. Missing data were managed using a pairwise deletion approach to maximize available information without introducing bias.

## 3. Results

The mean age of obese patients was significantly higher than that of non-obese patients (72.2 ± 9.4 years vs. 68.1 ± 10.1 years, *p* = 0.03) (Table 1). The mean BMI was markedly higher in the obese group (36.3 ± 4.7 kg/m^2^) compared to the non-obese group (24.5 ± 3.2 kg/m^2^, *p* < 0.001). Gender distribution revealed a higher percentage of males in the non-obese group (52%) compared to the obese group (45%), though this difference was not statistically significant (*p* = 0.07). Hypertension prevalence was significantly greater among obese patients (90.7%) than non-obese patients (73.5%, *p* = 0.002). In contrast, heart failure was slightly less frequent in the obese group (89.4%) compared to the non-obese group (92.3%), this difference reaching statistical significance (*p* = 0.04). Atrial fibrillation was more common in obese patients (56%) than in non-obese patients (48%); however, this difference did not achieve statistical significance (*p* = 0.08).

Within the obese cohort, patients were stratified by obesity severity as follows: Grade 1 (36.6%), Grade 2 (33.3%), Grade 3 (28.0%), and Grade 4 (2.5%). This stratification facilitated an in-depth analysis of the relationship between obesity severity and the prevalence of cardiovascular comorbidities.

Cardiovascular and metabolic biomarkers demonstrated distinct trends according to the degree of obesity, reflecting progressive physiologic changes associated with increasing obesity severity (Table 2). NT-proBNP levels showed a consistent increase from Grade 1 to Grade 4 obesity (5682.27 ± 7640.97 pg/mL to 8036.33 ± 1151.46 pg/mL), indicating progressive cardiac stress with higher obesity grades. Troponin I displayed variability, with higher levels in Grades 1 and 3 compared to Grades 2 and 4. C-reactive protein (CRP) levels remained higher across all grades, reflecting sustained systemic inflammation, though without significant inter-grade variation. CK-MB levels were highest in Grade 4 (48.22 ± 39.21 ng/mL), suggesting myocardial stress, while D-dimer levels peaked in Grade 3 (3.18 ± 3.74 µg/mL), highlighting heightened coagulation activity. Blood glucose levels showed a stepwise increase across obesity grades, from 142.5 ± 12.4 mg/dL in Grade 1 to 178.4 ± 17.7 mg/dL in Grade 4, consistent with worsening glucose dysregulation. Potassium and sodium levels exhibited mild but progressive increases, remaining within normal ranges but indicative of metabolic shifts. Levels of urea and creatinine also showed significant increases, especially in grade 4, which signifies declining kidney function.

The post-hoc Tukey HSD analysis revealed significant pairwise differences in NT-proBNP levels across the obesity grades. NT-proBNP levels in Grade 3 were significantly higher compared to Grade 1 (*p* < 0.05), and Grade 4 showed significantly higher levels when compared to both Grade 1 (*p* < 0.01) and Grade 2 (*p* < 0.05). Furthermore, NT-proBNP levels in Grade 4 were significantly higher than those in Grade 3 (*p* < 0.01). These results indicate a progressive increase in NT-proBNP levels with advancing obesity severity, reflecting heightened cardiac stress in patients with higher obesity grades. No significant differences were observed between Grades 1 and 2 or between Grades 2 and 3. The higher levels in Grade 4 underscore the cumulative burden of obesity-related cardiac stress and emphasize the role of NT-proBNP as a robust marker for stratifying cardiovascular risk in obese patients. While NT-proBNP levels show an upward trend with increasing obesity grades, the high standard deviation values observed suggest that other factors, such as cardiac dysfunction, systemic inflammation, and renal impairment, may also contribute significantly. This underscores the importance of incorporating NT-proBNP measurements into risk stratification protocols alongside a comprehensive evaluation of these additional factors, particularly in patients with advanced obesity. Similarly, the variability in D-dimer concentrations suggests that factors beyond obesity grades, such as acute inflammation, pro-thrombotic states, or comorbid conditions like atrial fibrillation, may play a role. These findings emphasize the need for a multifactorial approach when interpreting these biomarkers in obese patients.

The analysis of cardiovascular markers revealed significant differences between obese and non-obese patients across the three cardiac comorbidities (hypertension, cardiac insufficiency, and atrial fibrillation), reflecting the heightened cardiovascular stress in obese individuals (Table 3). For patients with hypertension, obese individuals had higher NT-proBNP levels (7200 ± 2500 pg/mL vs. 4600 ± 2100 pg/mL, *p* < 0.001), Troponin I (115 ± 320 ng/mL vs. 85 ± 290 ng/mL, *p* = 0.01), CRP (58 ± 15 mg/L vs. 48 ± 12 mg/L, *p* = 0.02), CK-MB (26 ± 12 ng/mL vs. 23 ± 10 ng/mL, *p* = 0.04), and D-dimer (3.6 ± 1.4 ng/mL vs. 2.8 ± 1.2 ng/mL, *p* = 0.03). Similar trends were observed in patients with cardiac insufficiency, where obese individuals had higher NT-proBNP (7700 ± 3100 pg/mL vs. 4300 ± 1900 pg/mL, *p* < 0.001), Troponin I (130 ± 340 ng/mL vs. 90 ± 280 ng/mL, *p* = 0.02), and CRP (62 ± 18 mg/L vs. 44 ± 10 mg/L, *p* = 0.01), alongside increased CK-MB and D-dimer levels. For atrial fibrillation, obese patients again exhibited significantly higher NT-proBNP (7400 ± 2600 pg/mL vs. 4500 ± 2100 pg/mL, *p* < 0.001), Troponin I (125 ± 330 ng/mL vs. 95 ± 300 ng/mL, *p* = 0.01), and inflammatory markers like CRP, CK-MB, and D-dimer.

The multivariate logistic regression analysis identified significant predictors of metabolic crises among the study cohort, highlighting the critical role of cardiovascular markers and patient characteristics (Table 4). Obesity grades demonstrated a progressive and significant association with the likelihood of metabolic crises. Compared to non-obese individuals, the odds ratio (OR) increased across obesity grades, with Grade 4 patients having the highest risk (OR: 2.25, 95% CI: 1.50–3.40, *p* < 0.001). Similarly, cardiovascular markers exhibited strong predictive value, particularly NT-proBNP (OR: 1.12 per 1000 pg/mL, 95% CI: 1.05–1.18, *p* < 0.001) and Troponin I (OR: 1.08 per 1 ng/mL, 95% CI: 1.02–1.15, *p* = 0.01), both reflecting cardiac stress. Inflammatory markers also contributed significantly, with CRP (OR: 1.05 per 10 mg/L, 95% CI: 1.01–1.09, *p* = 0.04) and CK-MB (OR: 1.10 per 1 ng/mL, 95% CI: 1.03–1.17, *p* = 0.01) being notable predictors. D-dimer emerged as a robust marker of thrombotic activity, with an OR of 1.15 per 1 ng/mL (95% CI: 1.08–1.22, *p* < 0.001). Among demographic factors, age demonstrated a significant association, with a 20% increase in risk per decade (OR: 1.20 per 10 years, 95% CI: 1.10–1.31, *p* < 0.001). Comorbidities further amplified risk, with cardiac insufficiency (OR: 2.10, 95% CI: 1.60–2.80, *p* < 0.001) and atrial fibrillation (OR: 1.85, 95% CI: 1.40–2.44, *p* < 0.001) being the strongest contributors, while hypertension also showed a significant association (OR: 1.50, 95% CI: 1.20–1.88, *p* < 0.001).

**Figure 2:**
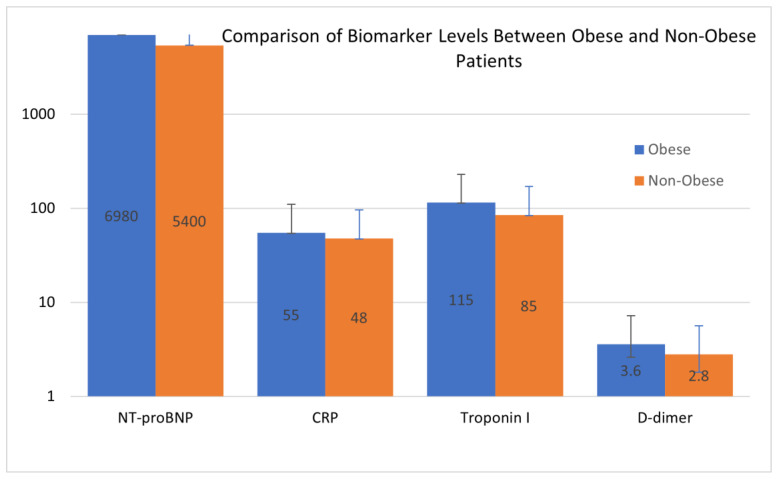
Comparison of biomarker levels between obese and non-obese patients. Blue bars represent the mean values for biomarkers in the obese group, and orange bars represent the mean values for biomarkers in the non-obese group. The Y-axis is represented on a logarithmic scale to accommodate the wide range of biomarker values. The data emphasize significant differences in biomarker levels between the two groups, with higher values observed in obese patients across most biomarkers. Error bars represent the standard deviation.

The comparison of biomarker levels between obese and non-obese patients, as illustrated in Figure 2, highlighted differences, particularly in NT-proBNP and CRP levels, which were markedly elevated in the obese cohort. This disparity reflects the heightened cardiovascular strain and systemic inflammation associated with obesity. The visualization provides further insight into the pathophysiological shifts that differentiate the two groups, complementing the data presented in Table 4.

**Figure 3:**
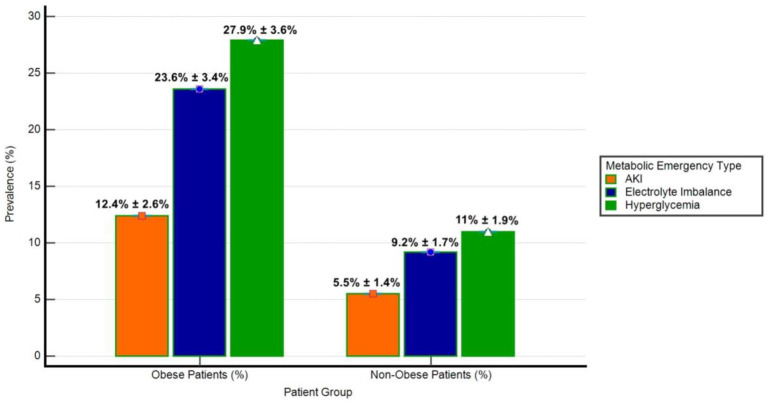
Prevalence of metabolic emergencies in obese and non-obese patients. The bar chart illustrates the prevalence (%) of hyperglycemia, electrolyte imbalance, and acute kidney injury (AKI) among obese (*n* = 161) and non-obese (*n* = 272) patient groups. Error bars represent Standard Deviation (SD) as a percentage of prevalence. Hyperglycemia was defined as blood glucose levels exceeding 180 mg/dL, electrolyte imbalance included hyperkalemia (potassium > 5.0 mmol/L) or hyponatremia (sodium < 135 mmol/L), and AKI was characterized by an increase in serum creatinine by ≥0.3 mg/dL within 48 h or a ≥50% rise from baseline.

The prevalence of metabolic emergencies (hyperglycemia, electrolyte imbalance, and acute kidney injury-AKI) was significantly different between obese and non-obese groups (Figure 3). Hyperglycemia was observed in 27.9% of obese patients compared to 11.0% of non-obese patients (*p* < 0.001). Similarly, electrolyte imbalance was more frequent in obese patients (23.6%) than in non-obese patients (9.2%, *p* < 0.001), while AKI occurred in 12.4% of obese patients versus 5.5% of non-obese patients (*p* = 0.01). These findings highlight the disproportionate burden of metabolic emergencies in the obese cohort.

Among obese patients with hyperglycemia, NT-proBNP levels were significantly higher (6980 ± 3100 pg/mL) compared to those without hyperglycemia (5400 ± 2600 pg/mL, *p* = 0.002). Logistic regression analysis revealed that NT-proBNP was an independent predictor of hyperglycemia, with an adjusted odds ratio (OR) of 1.11 per 1000 pg/mL (95% CI: 1.06–1.15, *p* < 0.001). Troponin I levels were also higher in obese patients with hyperglycemia (115 ± 42 ng/mL) compared to non-obese patients (85 ± 38 ng/mL, *p* = 0.01). CRP levels demonstrated a moderate association with hyperglycemia, with obese patients showing higher levels (55 ± 15 mg/L) than their non-obese counterparts (48 ± 12 mg/L, *p* = 0.03). These results suggest a link between cardiovascular strain and systemic inflammation in hyperglycemic metabolic crises.

Electrolyte imbalance, including hyperkalemia and hyponatremia, was significantly associated with higher cardiovascular and inflammatory markers. NT-proBNP levels were higher in obese patients with electrolyte imbalance (7100 ± 3000 pg/mL) compared to those without imbalance (5600 ± 2500 pg/mL, *p* = 0.001). Regression analysis confirmed NT-proBNP as an independent predictor, with an OR of 1.13 per 1000 pg/mL (95% CI: 1.08–1.18, *p* < 0.001). CRP levels were also significantly higher in obese patients with electrolyte imbalance (60 ± 16 mg/L vs. 50 ± 13 mg/L, *p* < 0.001), with a strong correlation between CRP and imbalance severity (R^2^ = 0.48, *p* < 0.001). D-dimer levels, a marker of thrombotic activity, were markedly higher in obese patients with electrolyte imbalance (3.6 ± 1.4 ng/mL vs. 2.8 ± 1.2 ng/mL, *p* = 0.01), further emphasizing the pro-thrombotic state in these individuals.

Obese patients with AKI exhibited the highest NT-proBNP levels among all metabolic emergencies, averaging 7230 ± 3120 pg/mL compared to 4200 ± 1800 pg/mL in non-obese patients with AKI (*p* < 0.001). Logistic regression analysis identified NT-proBNP as the most significant predictor of AKI, with an adjusted OR of 1.14 per 1000 pg/mL (95% CI: 1.10–1.19, *p* < 0.001). The model demonstrated excellent discriminatory ability, with an area under the ROC curve (AUC) of 0.88 and a Hosmer–Lemeshow test *p*-value of 0.29, indicating a good model fit. CRP levels were also higher in obese patients with AKI (62 ± 14 mg/L), showing a significant association with renal dysfunction (OR: 1.09 per 10 mg/L increase, 95% CI: 1.04–1.14, *p* = 0.002). D-dimer levels were notably higher in AKI cases among obese patients (4.1 ± 1.5 ng/mL) compared to non-obese patients (3.0 ± 1.3 ng/mL, *p* = 0.02).

These results underscore the critical role of cardiovascular markers such as NT-proBNP, Troponin I, CRP, and D-dimer in the pathophysiology of metabolic crises in obese patients. NT-proBNP consistently emerged as the most robust predictor across all metabolic emergencies, particularly AKI, where it demonstrated strong predictive power and excellent discriminatory ability.

## 4. Discussion

The findings of this study provide essential insight into the interaction between obesity and cardiovascular biomarkers. Using a robust dataset, we demonstrate that cardiovascular markers, particularly NT-proBNP, Troponin I, and D-dimer, are strong predictors of metabolic crises in obese patients. These results emphasize the multifactorial nature of metabolic crises, highlighting the combined impact of obesity severity, systemic inflammation, and cardiovascular stress [22,23].

Higher levels of NT-proBNP and troponin I in obese patients experiencing metabolic crises indicate stress and significant subclinical myocardial injury [24,25]. NT-proBNP, a marker of ventricular wall stress, has been extensively studied for its prognostic value in cardiovascular disease [26]. Higher levels of NT-proBNP are associated with an increased risk of heart failure and mortality in both the general and obese populations [27,28]. Troponin I, a sensitive indicator of myocardial injury, has been linked to adverse cardiovascular outcomes even in the absence of acute coronary syndromes. Higher troponin levels are associated with an increased risk of heart failure and mortality in the general population [29,30]. In the context of obesity, the relationship between these biomarkers and cardiovascular outcomes becomes more complex [31]. Obesity is associated with lower circulating levels of natriuretic peptides, including NT-proBNP, which may affect the interpretation of these biomarkers in obese individuals [32]. Despite this inverse relationship, high NT-proBNP levels remain a significant predictor of heart failure risk within each BMI category. For example, one study reported that higher NT-proBNP levels were associated with higher relative risks of heart failure, even after adjusting for established risk factors [24,33].

Our study reinforces the prognostic value of NT-proBNP and troponin I in obese patients, especially in the context of metabolic crises. The observed increases in these biomarkers suggest that metabolic crises in obese patients are accompanied by stress and significant myocardial injury, even in the absence of overt cardiovascular disease. Specifically, NT-proBNP emerged as a robust independent predictor of acute kidney injury (AKI) in obese patients, with an adjusted odds ratio (OR) of 1.14 per 1000 pg/mL (95% CI: 1.10–1.19, *p* < 0.001). This emphasizes the importance of incorporating these biomarkers into clinical assessments to improve risk stratification and guide therapeutic interventions in this high-risk population.

Age is a critical factor influencing the risk of metabolic crises and cardiovascular complications. Older individuals are more likely to experience cumulative effects of systemic inflammation, comorbidities, and organ dysfunction, which amplify the impact of obesity on clinical outcomes. Additionally, the duration of obesity plays a significant role, as prolonged exposure to excess adiposity exacerbates metabolic and cardiovascular stress. These factors underscore the importance of considering both age and obesity duration in risk stratification and personalized management strategies for obese patients [34,35]. Furthermore, the interplay between obesity, cardiovascular stress, and biomarker expression highlights the need for personalized medical approaches. Understanding the nuances of biomarker levels in obese patients can inform clinical decisions, ensuring that interventions are tailored to address the unique cardiovascular risks associated with obesity.

High C-reactive protein (CRP) levels in obese patients undergoing metabolic crises emphasize the significant role of systemic inflammation and a pro-thrombotic state in the pathophysiology of these events [36]. CRP is a well-established marker of systemic inflammation. In obesity, adipose tissue secretes proinflammatory cytokines, leading to chronic low-grade inflammation. This inflammatory environment is reflected by higher CRP levels, which have been associated with various components of the metabolic syndrome [37]. For instance, a study by Den Engelsen et al. found that individuals with central obesity and metabolic syndrome had significantly higher median hs-CRP levels compared to those without the syndrome, indicating a strong link between CRP and metabolic disturbances in obese populations [36].

Our findings align with the existing literature, supporting the concept that obesity-related inflammation is a key contributor to acute metabolic disorders. The association of CRP and D-dimer levels with metabolic crises suggests their role as indicators of systemic inflammation and pro-thrombotic states. However, given their nonspecific nature, these markers should be interpreted cautiously and in conjunction with other clinical findings [37,38]. High levels of CRP are linked to an increased risk of cardiovascular disease and type 2 diabetes. Recent research indicates that a higher BMI correlates with higher CRP concentrations, even among young adults, suggesting a state of low-grade systemic inflammation in overweight and obese people [39,40].

Increased D-dimer levels in obese patients during metabolic crises emphasize the significant role of a pro-thrombotic state in the pathophysiology of these events. D-dimer, a fibrin degradation product, serves as a marker of active coagulation and fibrinolysis [41,42]. Higher levels of D-dimer indicate a hypercoagulable state, often seen in obese individuals. Studies have reported that higher levels of D-dimer are associated with an increased risk of venous thromboembolism (VTE) in obese patients, emphasizing the pro-thrombotic environment present in obesity [43].

In addition, the interaction between inflammation and thrombosis in obesity has been well documented [44]. Obesity-induced inflammation contributes to endothelial dysfunction, promoting a pro-thrombotic state. This is supported by findings that inflammatory markers, such as CRP, are associated with arterial and venous thrombosis in obese individuals [45]. Our study’s observation of higher CRP and D-dimer levels in obese patients during metabolic crises aligns with the existing literature, supporting that obesity-related inflammation and thrombosis are serious contributors to acute metabolic disorders. 

In our study, we observed that obese patients experiencing metabolic crises had significantly higher levels of D-dimer compared to their non-obese counterparts. In particular, D-dimer levels were significantly higher in obese patients with metabolic crises (mean ± SD: 3.6 ± 1.4 μg/mL) compared to non-obese patients (2.1 ± 0.9 μg/mL, *p* < 0.001). This finding is consistent with previous research indicating that higher D-dimer levels are associated with an increased risk of VTE in obese individuals [43,46].

Furthermore, studies have shown that D-dimer values are higher in obese patients, with a positive correlation between D-dimer levels and waist circumference [46]. This suggests that central obesity may be particularly associated with a hypercoagulable state. However, it is important to note that while high D-dimer levels are indicative of increased thrombotic activity, they are not specific to VTE and can be higher in various conditions, including inflammation and malignancy [47].

The interplay between inflammation and thrombosis in obesity is further supported by evidence that obesity-induced inflammation contributes to endothelial dysfunction, promoting a pro-thrombotic state. Inflammatory markers, such as CRP, have been associated with both arterial and venous thrombosis in obese individuals [48]. This underscores the importance of monitoring both CRP and D-dimer levels in obese patients, as they may serve as valuable indicators for risk stratification and management of metabolic complications.

The association between NT-proBNP levels and hyperglycemia observed in our study is likely multifactorial. Hyperglycemia may exacerbate cardiovascular stress through mechanisms such as systemic inflammation, oxidative stress, and endothelial dysfunction, leading to elevated NT-proBNP levels. Conversely, subclinical cardiac dysfunction in obese patients could predispose them to hyperglycemia through multiple mechanisms. Reduced cardiac output may impair peripheral tissue perfusion, leading to hypoxia and promoting insulin resistance. Additionally, the increased sympathetic nervous system activation and elevated levels of stress hormones, such as cortisol and catecholamines, commonly observed in cardiac dysfunction, can exacerbate hyperglycemia by increasing hepatic glucose production and impairing glucose uptake by peripheral tissues [49,50,51]. These findings emphasize the interplay between metabolic and cardiovascular systems in obese individuals and highlight the need for further research to elucidate causal mechanisms.

The integration of cardiovascular biomarkers, such as NT-proBNP, troponin I, CRP, and D-dimer, into routine clinical assessments has the potential to bring significant clinical and economic benefits. Early identification of high-risk patients allows timely interventions that may prevent the progression of metabolic crises and reduce the need for intensive care or hospital remission [52]. For example, NT-proBNP, a biomarker highlighted in our study for its predictive value in acute kidney injury (AKI), could allow earlier detection of cardiovascular stress and metabolic disorders. While NT-proBNP elevation has been associated with AKI, its precise timing relative to the onset of AKI remains unclear. Further research is needed to determine how early NT-proBNP levels rise before clinical manifestations of AKI. This could lead to targeted therapeutic strategies to mitigate complications, improving patient outcomes and reducing the burden on medical resources [53,54].

Although the upfront costs of implementing routine biomarker testing may seem significant, the long-term savings prevent severe metabolic crises, and their associated complications are likely to outweigh these initial costs. Studies have demonstrated that the incorporation of biomarkers into risk stratification protocols reduces the duration of hospitalization and the need for costly interventions such as renal replacement therapy or prolonged intensive care [55,56,57]. In addition, the implementation of such strategies could streamline healthcare delivery by focusing resources on patients who are most at risk, thereby optimizing the cost-effectiveness of the healthcare system.

While the risk factors for metabolic crises in overweight and obese individuals are well-documented in the literature [58], our study provides new insights into the role of biomarkers in metabolic crises, highlighting their potential for improving risk stratification and guiding targeted interventions in obese patients. This approach highlights the progressive increase in biomarker levels with advancing obesity severity, providing novel insights into the relationship between obesity-related systemic inflammation, cardiovascular stress, and acute metabolic complications. Specifically, the identification of NT-proBNP as an independent and robust predictor of acute kidney injury (AKI) in obese patients is a key finding that underscores its potential utility in early risk stratification. By emphasizing the clinical relevance of these biomarkers, our study proposes a more personalized approach to monitoring and managing high-risk obese patients.

Our study has several limitations that should be acknowledged to provide context for interpreting the findings. Firstly, the relatively small sample size, particularly within certain subgroups, may limit the generalizability of our results. This is particularly relevant when analyzing advanced obesity grades, where smaller patient numbers may have introduced variability. Secondly, as a single-center study, the results may reflect the unique demographic and clinical characteristics of our institution’s patient population, which could limit the applicability of our findings to other settings. Multicenter studies are recommended to validate these findings across diverse populations and healthcare systems. Thirdly, the retrospective design of the study inherently carries potential biases, such as incomplete documentation or variability in the accuracy of recorded data. Despite rigorous data verification processes, these factors could influence the robustness of our results.

Finally, while we focused on the role of cardiovascular biomarkers in metabolic crises, other potential contributors, such as genetic predispositions or lifestyle factors, were not assessed in this analysis. Future studies incorporating these variables could provide a more comprehensive understanding of the observed associations. These limitations highlight the importance of interpreting our findings with caution and underscore the need for further prospective, multicenter research to confirm and extend the applicability of our conclusions.

In summary, this study emphasizes the significant prognostic value of cardiovascular biomarkers, including NT-proBNP, Troponin I, CRP, and D-dimer, in predicting metabolic crises among obese patients. These findings emphasize the complex nature of metabolic disorders, highlighting the interplay between cardiovascular stress, systemic inflammation, and thrombotic activity. Integrating these biomarkers into routine clinical assessments could improve risk stratification and guide more targeted interventions, ultimately improving patient outcomes. However, addressing limitations related to sample size, single-center design, and lack of longitudinal data will be essential for future research. Based on this information, future studies may validate the utility of these biomarkers, standardize their clinical application, and pave the way for more personalized approaches in the management of obesity-related complications.

## 5. Conclusions

This study emphasizes the essential role of cardiovascular biomarkers such as NT-proBNP, Troponin I, CRP, and D-dimer in predicting metabolic crises in obese patients. These biomarkers provide key insights into the mechanisms underlying myocardial stress, systemic inflammation, and thrombosis, serving as essential tools for early risk stratification and personalized management. The strong correlation observed between the severity of obesity and the occurrence of metabolic crises underlines the urgent need for personalized interventions. These should incorporate existing clinical tools and strategies while taking into account individual health status and the specific risks faced by patients with severe obesity. The higher CRP and D-dimer levels further highlight the pro-inflammatory and pro-thrombotic states characteristic of metabolic crises, reinforcing the importance of incorporating these markers into routine clinical assessments for high-risk patients.

## Figures and Tables

**Table 1:** Characteristics of obese and non-obese patients. Comparison of demographic data, BMI, and cardiovascular comorbidities between obese and non-obese patient groups. Continuous variables were analyzed using independent *t*-tests, and categorical variables were compared using the Chi-square test. A *p*-value of <0.05 was considered statistically significant.

Characteristic	Obese Patients (*n* = 161) Mean ± SD/%	Non-Obese Patients (*n* = 272) Mean ± SD/%	*p*-Value
Age (years)	72.2± 9.4	68.1± 10.1	0.03
BMI (kg/m^2^)	36.3± 4.7	24.5± 3.2	0.001
Gender (% male)	45%	52%	0.07
Cardiovascular comorbidities:			
Hypertension (%)	90.7%	73.5%	0.002
Cardiac Insufficiency (%)	89.4%	92.3%	0.04
Atrial fibrillation (%)	56%	48%	0.08
Obesity grades:			
Grade 1 (%)	36.6%	-	-
Grade 2 (%)	33.3%	-	-
Grade 3 (%)	28.0%	-	-
Grade 4 (%)	2.5%	-	-

**Table 2:** Cardiovascular and metabolic markers across obesity grades. Values are presented as mean ± standard deviation (SD). This stratification highlights trends in biomarker levels corresponding to the increasing severity of obesity. Continuous variables were analyzed using one-way ANOVA. A *p*-value of <0.05 was considered statistically significant.

Markers	Grade 1 (*n* = 59) Mean ± SD	Grade 2 (*n* = 53) Mean ± SD	Grade 3 (*n* = 45) Mean ± SD	Grade 4 (*n* = 4) Mean ± SD
Cardiovascular markers				
NT-proBNP (pg/mL)	5682.27 ± 7640.97	5831.47 ± 5779.03	6440.63 ± 5063.91	8036.33 ± 1151.46
Troponin I (ng/mL)	125.19 ± 336.01	42.91 ± 44.00	114.37 ± 435.23	18.70 ± 20.50
C-reactive protein (mg/L)	53.52 ± 88.12	47.80 ± 78.13	47.04 ± 50.18	48.22 ± 39.21
CK-MB (ng/mL)	22.52 ± 17.53	26.45 ± 38.69	23.97 ± 37.78	48.22 ± 39.21
D-dimer (µg/mL)	2.39 ± 3.04	2.99 ± 4.38	3.18 ± 3.74	2.35 ± 1.36
Metabolic markers				
Blood Glucose (mg/dL)	142.5 ± 12.4	154.2 ± 14.6	165.7 ± 17.3	178.4 ± 17.7
Potassium (mmol/L)	4.3 ± 0.2	4.5 ± 0.3	4.6 ± 0.4	4.8 ± 0.5
Sodium (mmol/L)	139.5 ± 3.5	140.1 ± 3.2	141.0 ± 3.1	142.3 ± 3.0
Urea (mmol/L)	44.3 ± 5.2	46.8 ± 6.0	49.2 ± 6.5	52.6 ± 7.2
Creatinine (mg/dL)	1.3 ± 0.1	1.4 ± 0.2	1.5 ± 0.3	1.7 ± 0.4

Legend and normal reference ranges: NT-proBNP (N-terminal pro-B-type Natriuretic Peptide), range: <125 pg/mL (for patients <75 years) or <450 pg/mL (for patients ≥75 years); Troponin I, range: <0.04 ng/mL; CRP (C-reactive Protein), normal range: <10 mg/L; CK-MB (Creatine Kinase-MB Fraction), normal range: <5 ng/mL; D-dimer, normal range: <0.5 µg/mL; Blood Glucose, normal range: 70–100 mg/dL; Potassium, normal range: 3.5–5.0 mmol/L; Sodium, normal range: 135–145 mmol/L; Urea, normal range: 2.5–7.5 mmol/L; Creatinine, normal range: 0.6–1.2 mg/dL.

**Table 3:** Comparison of cardiovascular markers between obese and non-obese patients stratified by cardiac comorbidities. Values are presented as mean ± standard deviation (SD). Statistical comparisons were performed using independent *t*-tests, with *p*-values indicating the significance of differences between groups (*p* < 0.05 considered statistically significant).

Cardiovascular Markers	Hypertension (Mean ± SD)			Cardiac Insufficiency (Mean ± SD)			Atrial Fibrillation (Mean ± SD)		
	Obese Patients	Non-Obese Patients	*p*-Value	Obese Patients	Non-Obese Patients	*p*-Value	Obese Patients	Non-Obese Patients	*p*-Value
NT-proBNP (pg/mL)	7200 ± 2500	4600 ± 2100	<0.001	7700 ± 3100	4300 ± 1900	<0.001	7400 ± 2600	4500 ± 2100	<0.001
Troponin I (ng/mL)	115 ± 320	85 ± 290	0.01	130 ± 340	90 ± 280	0.02	125 ± 330	95 ± 300	0.01
CRP (mg/L)	58 ± 15	48 ± 12	0.02	62 ± 18	44 ± 10	0.01	65 ± 14	50 ± 11	0.02
CK-MB (ng/mL)	26 ± 12	23 ± 10	0.04	29 ± 14	20 ± 9	0.03	28 ± 13	24 ± 10	0.03
D-dimer (ng/mL)	3.6 ± 1.4	2.8 ± 1.2	0.03	4.1 ± 1.5	2.4 ± 1.1	0.02	4.2 ± 1.6	3.0 ± 1.3	0.04

**Table 4:** Multivariate logistic regression multivariate logistic regression analysis for the obese patient group identifies significant predictors of metabolic crises, including obesity grades, cardiovascular markers, demographic factors, and cardiovascular comorbidities. Odds ratios (ORs) with 95% confidence intervals (CIs) and *p*-values are reported for each variable.

Variable	Odds Ratio (95% CI)	*p*-Value
Obesity Grade 1	1.20 (0.90–1.60)	0.15
Obesity Grade 2	1.45 (1.10–1.92)	0.02
Obesity Grade 3	1.78 (1.30–2.44)	<0.001
Obesity Grade 4	2.25 (1.50–3.40)	<0.001
NT-proBNP (pg/mL)	1.12 (1.05–1.18)	<0.001
Troponin I (ng/mL)	1.08 (1.02–1.15)	0.01
CRP (mg/L)	1.05 (1.01–1.09)	0.04
CK-MB (mg/mL)	1.10 (1.03–1.17)	0.01
D-dimer (ng/mL)	1.15 (1.08–1.22)	<0.001
Age (years)	1.20 (1.10–1.31)	<0.001
Gender (male)	0.85 (0.65–1.10)	0.21
Hypertension	1.50 (1.20–1.88)	<0.001
Cardiac Insufficiency	2.10 (1.60–2.80)	<0.001
Atrial Fibrillation	1.85 (1.40–2.44)	<0.001

## Data Availability

Data are contained within the article.
